# Artificial Intelligence for Prediction and Detection of Atrial Fibrillation from Sinus-Rhythm Electrocardiograms and Ambulatory Monitoring

**DOI:** 10.3390/biomedicines14051058

**Published:** 2026-05-07

**Authors:** Panteleimon Pantelidis, Nikolaos Vythoulkas-Biotis, Athanasios Samaras, Panagiotis Theofilis, Raffaele De Lucia, Polychronis Dilaveris, Theodore G. Papaioannou, Evangelos Oikonomou, Gerasimos Siasos

**Affiliations:** 1 3rd Department of Cardiology, National and Kapodistrian University of Athens, 11527 Athens, Greece; vbnikos@gmail.com (N.V.-B.); hrodil1@yahoo.com (P.D.); boikono@med.uoa.gr (E.O.); gsiasos@med.uoa.gr (G.S.); 2 Medical School, Aristotle University of Thessaloniki, 54124 Thessaloniki, Greece; ath.samaras.as@gmail.com; 3 1st Department of Cardiology, National and Kapodistrian University of Athens, 11527 Athens, Greece; panos.theofilis@hotmail.com; 4 2nd Division of Cardiology, Cardiac Thoracic and Vascular Department, Azienda Ospedaliero Universitaria Pisana, 56124 Pisa, Italy; r.delucia.md@gmail.com; 5 Department of Biomedical Engineering, Medical School, National and Kapodistrian University of Athens, 11527 Athens, Greece; teogpap@gmail.com

**Keywords:** atrial fibrillation, arrhythmias, artificial intelligence, deep learning, machine learning, predictive modeling, sinus rhythm, electrocardiogram, monitoring

## Abstract

Atrial fibrillation (AF) is a highly prevalent arrhythmia associated with stroke, heart failure and excess mortality. Yet, “silent” AF episodes remain undetected, leading to underestimation of disease burden. Additionally, paroxysms occur in an “unpredictable” way, and available clinical scores only stratify long-term AF risk with moderate discrimination, lacking the ability to evaluate near-term events. Artificial intelligence (AI) applied to sinus rhythm from short or continuous electrocardiogram (ECG) recordings shows that such predictive information is hidden in “plain sight.” This complementary approach seeks to uncover latent AF substrate and forecast imminent AF episodes. Deep-learning models trained on 10-s, 12-lead ECGs can identify individuals with prevalent or long- or near-term AF with areas under the curve (AUCs) up to 0.90, outperforming established clinical scores. Image-based AI-ECG models extend these capabilities to paper or scanned ECGs. Furthermore, AI algorithms applied to 24-h Holter and multi-day patch recordings achieve AUCs ≥0.80 for detecting occult AF or predicting it within 14 days, consistently surpassing risk scores like C_2_HEST and HATCH. Short-term models utilizing heart-rate variability features further demonstrate that AF can be anticipated minutes to hours before onset, with accuracies around 90% in curated datasets. However, most AI-AF studies remain retrospective, single-system and focused on diagnostic yield rather than clinical outcomes like stroke or mortality. Moreover, few pragmatic trials have evaluated AI-guided AF screening and its translation into clinical benefit. Robust prospective trials and standardized evaluation frameworks are needed before AI-guided AF prediction can be routinely integrated into clinical decision-making.

## 1. Introduction

Atrial fibrillation (AF) is among the most prevalent arrhythmias globally and is associated with a two- to five-fold increased risk of stroke, heart failure, and all-cause mortality. Its incidence is rising with population aging, and more than 50 million individuals are estimated to be affected worldwide [1]. Many AF episodes are asymptomatic or paroxysmal, and therefore opportunistic or single-time-point electrocardiogram (ECG) screening can miss a substantial fraction of individuals who might benefit from anticoagulation [2]. Traditional extended rhythm monitoring with 24-h Holter or 14-day patches, and in selected cases, implantable loop recorders (ILRs), detects additional subclinical AF but is costly due to its low event rate, and the value of treating subclinical AF is still debatable [3]. Clinical scores such as HATCH and CHARGE-AF, and later C_2_HEST and Taiwan AF, have been developed to stratify AF onset or progression risk, but their discrimination remains limited [4,5,6,7,8]. Clinical scores can inform which patients should be screened but cannot resolve on their own who harbors an advanced atrial substrate likely to manifest AF in the near term. AI-based analysis of ECG seeks to fill this gap by decoding AF-related lesions and triggers reflected in ostensibly normal recordings.

The artificial intelligence (AI) revolution has also recently entered this field, shifting the goal from detecting overt AF to uncovering “silent” latent types or even predicting its onset from ECG records harvested from digital ECG archives and large-scale Holter and patch repositories. This enables AI algorithms to infer AF risk directly from raw sinus-rhythm ECG signals or from derived parameters, such as heart rate variability (HRV), with variable prediction horizons ranging from minutes to years, thus guiding targeted monitoring [9]. Since 2021, there has been a particular proliferation of relevant advances, spanning from generalizable image-based AI-ECG to single-lead patch-based home solutions predicting near-term or imminent AF and even efforts in pragmatic trials that rigorously test AI-guided AF risk screening [10]. At this point, it is important to distinguish such predictive approaches from the dominant real-world deployment of consumer wearable devices. Landmark studies, including the Apple Heart Study and the Fitbit Heart Study, have established smartwatches and other devices as tools for population-level AF screening [11,12]. However, their official utility currently lies in detecting concurrent, overt AF, typically via single-lead ECGs recorded during symptomatic episodes or photoplethysmography-detected rhythm irregularities. In contrast, the AI-ECG models at hand perform a fundamentally different task: uncovering hidden atrial substrates and predicting future or latent AF exclusively from periods of sinus rhythm. Consequently, while wearables represent a massive leap in arrhythmia detection, their current function places them outside the scope of sinus-rhythm AF prediction.

Collectively, all these efforts facilitate real-time monitoring and hold the potential to prevent adverse outcomes stemming from the sudden, asymptomatic onset of disease, changing the response from “re-active” to “pro-active” [13]. Furthermore, risk-individualized monitoring helps “map” the full landscape of AF burden, allowing clinicians to determine the potential benefits of prompt intervention. This narrative review summarizes contemporary AI approaches for both short- and long-term AF prediction from sinus-rhythm ECG segments and ambulatory recordings and attempts to unfold and explain the evolution in the field, provide a comprehensive update on prior efforts, and highlight convergent findings across horizons and modalities while outlining critical gaps and priorities for future research.

## 2. The Literature Search Strategy and Results

We employed a structured search approach to identify the relevant literature and ensure reproducibility, guided by the Preferred Reporting Items for Systematic Reviews and Meta-Analyses extension for Scoping Reviews (PRISMA-ScR) framework (Supplementary Materials) [14]. We systematically searched electronic databases (Medline/OVID, Embase/OVID, and Scopus) for articles published between the inception date of each source (1946, 1974, and 1970, respectively) and April 2026. The search utilized combinations and variations in keywords including “artificial intelligence”, “deep learning”, “machine learning”, “atrial fibrillation”, “electrocardiogram”, “sinus/normal rhythm”, and “prediction” (Supplementary Materials). We also applied snowballing by screening the reference lists of retrieved articles. We included peer-reviewed original research articles evaluating AI-based short-term and long-term AF prediction or latent AF detection from sinus-rhythm ECGs and ambulatory monitoring. We excluded non-peer-reviewed preprints and conference abstracts to maintain evidence reliability. Two authors independently assessed the retrieved articles for eligibility. In cases of disagreement, a third author was involved, and a final decision was reached by majority vote. From an initial yield of 459 titles, 32 studies were finally included in this review.

## 3. AI-Enabled ECG for Latent AF During Sinus Rhythm

**Figure 1:**
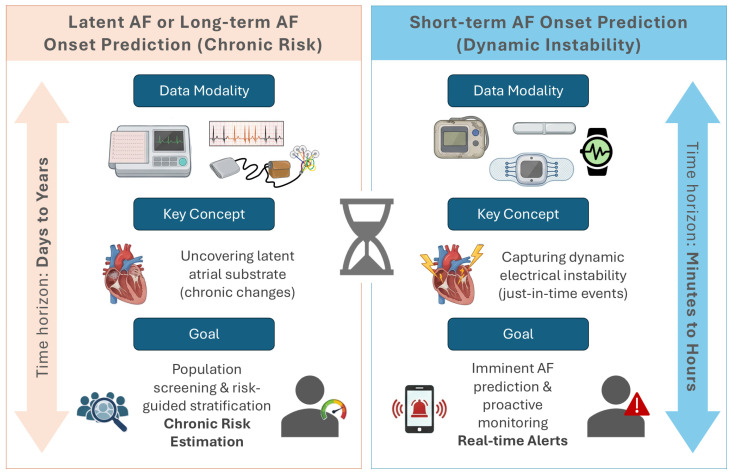
Conceptual framework and data modalities for AI-based AF prediction. The time-horizon continuum addresses both short-term forecasting (minutes to days) and long-term risk prediction (months to years). Modalities are mapped to their optimal predictive windows: continuous Holter and patch monitors, along with single-lead wearables, are predominantly utilized for imminent/short-term triggers, whereas standard 12-lead ECGs and ECG/EHR-based models are primarily deployed to capture long-term structural atrial substrate remodeling.

AI-based prediction of AF encompasses a heterogeneous family of approaches that differ in modality, exact prediction task, and time horizon. Some models analyze standard 10-s, 12-lead ECGs obtained in sinus rhythm to uncover a chronic atrial substrate associated with prevalent, latent, or long-term incident AF, essentially estimating an individual’s baseline propensity to develop AF over months to years [9,15,16,17]. Others focus on ambulatory signals, including Holter and patch recordings or beat-to-beat HRV, to detect occult AF within the same recording or to forecast episodes over short horizons ranging from minutes to hours, thereby capturing dynamic periods of electrical instability and trigger susceptibility [18,19,20,21]. These differences in modality and horizon dictate model architecture, labeling strategies, and potential clinical applications: long-term prediction speaks to substrate-guided risk stratification and screening, whereas short-term prediction targets proactive monitoring with just-in-time alerts (Figure 1).

### 3.1. Deep Learning on 12-Lead Sinus-Rhythm ECG

The seminal work by Attia and colleagues at Mayo Clinic demonstrated that an AI model, with a complex convolutional neural network architecture (CNN), trained on 649,931 10-s, 12-lead sinus-rhythm ECGs from 180,922 patients could identify individuals with prior or near-term AF (before or within 31 days of the ECG) with an area under the receiver operating characteristic curve (AUC) of 0.87 using a single ECG and approximately 0.90 when aggregating across multiple ECGs per patient. Sensitivity and specificity were each around 79% in the internal test set, and the model’s positive predictive value improved when requiring repeated high-risk predictions over several ECGs [22]. When interpreting these results, it is crucial to distinguish between the detection of concurrent, latent AF and the pure prediction of future incident AF. For instance, while the model achieved a remarkable performance, the ground truth for positives included individuals with at least one AF recording within 31 days of the sinus-rhythm ECG. This blurs the line between detecting latent AF and purely forecasting it. However, in practice, the notions of long-term onset prediction and latent AF detection often coincide. Unless a patient undergoes continuous monitoring via an implantable loop recorder or other cardiac implantable electronic device (CIED) from the time of the index sinus-rhythm ECG to the AF event, the possibility of preceding, asymptomatic paroxysmal episodes cannot be definitively excluded. Thus, long-term AI-ECG models inherently capture a blend of underlying substrate and undiagnosed paroxysms.

Subsequent analyses applied the same AI-ECG AF probability to population-based community cohorts without known AF at baseline and showed performance comparable to the well-established CHARGE-AF score. Higher AI-ECG AF probability was associated with increased long-term incidence of AF and ischemic stroke [17], with c-statistics around 0.69 for AF and a 3-fold risk when comparing the highest vs. the lowest probability quartiles. These findings supported the notion that the network captured a subclinical atrial cardiomyopathy phenotype that predisposes to AF.

Raghunath et al. extended this paradigm by training deep neural networks on 1.6 million ECG traces from 430,000 patients without prior AF to predict new-onset AF within one year [15]. Their model achieved an AUC of 0.85 for 1-year incident AF and showed a 7-fold difference in long-term AF risk between the highest and lowest risk deciles over 30-year follow-up. In a simulated deployment, targeting continuous monitoring to patients in the top risk decile yielded a number needed to screen of nine to detect one new AF case and identified approximately 62% of individuals who later experienced AF-related stroke. Other groups have developed and validated similar 12-lead AI-ECG models. Baek et al. reported a deep-learning algorithm trained on sinus-rhythm ECGs that achieved test-set AUCs around 0.75 for discriminating paroxysmal AF from controls [16], while Brant and colleagues conducted a multinational study using routine clinical ECGs and demonstrated that AI-ECG scores predict incident AF with c-statistics near 0.80 across geographically diverse cohorts, with incremental prognostic value over CHARGE-AF [23].

Collectively, these works demonstrate that relatively short resting ECGs contain sufficient information to characterize an underlying atrial substrate that substantially elevates AF risk (Table 1). In contrast to conventional risk scores, AI-ECG models achieve this without explicit manual input of age or comorbidities. However, biological age has been shown to be deeply encoded within ECG morphology and may indirectly represent a primary driver of model predictions [24,25].

### 3.2. Serial, Feature- and Image-Based AI-ECG

While end-to-end CNNs involving raw ECG waveforms as inputs can provide strong discrimination, they are often criticized for limited interpretability. Choi et al. approached AF prediction by extracting detailed P-wave, PR, QRS, and T-wave features; heart-rate variability indices; and further features from serial ECGs, then using AI models to predict new-onset AF based on temporal changes. Serial-ECG models consistently outperformed single-time-point models, supporting the concept that progressive atrial remodeling over months to years contributes information beyond static ECG morphology. Feature-importance analyses in such models reinforced the importance of P-wave characteristics but crucially revealed that the model heavily relied on the patient’s age for its predictions, confirming that age dominates the algorithmic weighting, possibly functioning as a confounder and diminishing the importance of other predictors substantially [26].

A practical limitation of waveform-based AI-ECG is the requirement for digital ECG signals. Zeidaabadi and colleagues addressed this by training image-based CNNs on scanned or PDF ECGs to predict incident AF, achieving c-statistics around 0.72–0.75, and demonstrating improved discrimination when combining AI-image scores with CHARGE-AF [27]. Gadaleta’s at-home patch study similarly used derived single-lead ECG segments but showed that even relatively noisy ambulatory signals could support strong near-term prediction [18].

### 3.3. External Validation and Prospective Deployment

While many studies show promising internal results, few undergo external validation to confirm broad and even inter-ethnic generalizability. Recently, multinational validations have begun to emerge, demonstrating that AI-ECG models can preserve discrimination across diverse cohorts, including Korean, Brazilian, UK, and other populations, often providing incremental prognostic value over clinical scores [23,28]. In this direction, Christopoulos et al. performed a population-based validation of the Mayo AI-ECG AF-risk model and found that AI-ECG probabilities predicted incident AF with c-statistics around 0.69 [17], while Lee et al. have examined inter-ethnic generalizability by testing AI-ECG models on a multi-ethnic CODE 15% cohort, showing that discrimination is preserved [29]. However, testing models on multi-ethnic cohorts remains the exception rather than the rule. Beyond retrospective validations [17,30], the pragmatic trial by Noseworthy and colleagues provides the first real-world test of AI-guided AF screening [31]. In that study, more than 669,000 patients with routine ECGs were retrospectively scored using an AI-ECG AF-risk model; 1003 older adults with elevated stroke risk and no known AF were then enrolled into a decentralized trial and offered up to 30 days of continuous patch monitoring. AF (≥30 s) was detected in 7.6% of AI-high-risk participants vs. 1.6% of AI-low-risk participants, corresponding to an odds ratio of approximately 5. AF diagnosis at around 10 months was 10.6% vs. 3.6% in AI-high-risk screened participants vs. matched usual-care controls (*p* < 0.0001). These findings suggest that AI-ECG can meaningfully enrich the monitored population, revealing up to now “hidden” AF cases, while at the same time reducing the cost of extended monitoring. However, this impressive diagnostic yield must be carefully contextualized. The trial utilized a non-randomized design, with the baseline population already at elevated risk for stroke and AF. Furthermore, the lack of “hard” clinical outcomes (stroke/mortality) limits conclusions on actual clinical benefit, leaving unanswered whether AI-guided screening improves hard outcomes beyond increased AF detection. Current guidelines, therefore, still regard AI as promising but investigational in AF when it comes to decision-making [32].

**Table 1:** Summary of representative studies developing or evaluating artificial intelligence models for the detection and prediction of atrial fibrillation from electrocardiogram signals in sinus rhythm.

Study	Study Design and Population	Data Modality	Outcome and Time Horizon	Modeling Approach	Key Performance and Calibration Metrics
Long-term onset or latent AF					
Alreshidi et al. 2024 [33]	Retrospective multi-institution ECG datasets across several centers	Multi-lead short ECG segments	Early AF prediction	Federated CNN-LSTM (Fed-CL) trained across clients	Reported strong discrimination with internal-test AUC ≈ 0.94 while preserving data privacy
Attia et al. 2019 [22]	180,922 pts, retrospective single US health-system ECG database	10 s 12-lead sinus-rhythm ECG	Latent/near-term AF (prior AF or AF ≤ 30 days)	End-to-end CNN on raw ECG	Single ECG AUC of 0.87; sensitivity of 79.0%; specificity of 79.5%; multiple ECGs per patient AUC ≈ 0.90 for AF identification
Baek et al. 2021 [16]	Hospital retrospective cohort without AF on index ECG	12-lead sinus-rhythm ECG	Prevalent paroxysmal AF and future AF	RNN (LSTM) on 12-lead ECG	AUC of 0.79 (internal) and 0.75 (external) for AF during sinus rhythm
Brant et al. 2025 [23]	Multinational community, retrospective cohorts with routine ECGs	Routine 12-lead clinical ECGs	Incident AF over multi-year follow-up	DL-ECG risk model (CNN)	C-statistics ≈ 0.80 for incident AF across cohorts; incremental to clinical scores; ICI of 0.007–0.032; Brier scores of 0.011–0.037
Cho et al. 2025 [28]	Korean, UK, and US retrospective population cohorts	12-lead ECG in sinus rhythm	Incident/early-onset AF; association with “ECG-age” gap	ResNet-type age-prediction network; age gap as a biomarker	AI-ECG age ≥7 y older than chronological age associated with ≈2–3-fold higher AF risk across cohorts
Choi et al. 2024 [26]	Serial ECG retrospective cohort with repeated 12-lead ECGs	P-QRS-T features and HRV indices from serial 12-lead ECGs	Incident AF based on temporal left-atrial remodeling	Feature engineering + ML (e.g., gradient boosting, tree-based models)	Serial-ECG models outperformed single-ECG models; C-statistics ≈0.80–0.88 for incident AF
Christopoulos et al. 2020 [17]	Population-based, retrospective cohort without AF at baseline	12-lead sinus-rhythm ECG	Incident AF over long-term follow-up	Application of Attia AI-ECG probability in Cox models	AI-ECG AF probability C-statistic ≈0.69 for incident AF, comparable to CHARGE-AF
Dupulthys et al. 2024 [34]	68,880 pts from a retrospective hospital dataset (AZ Delta hospital)	10-s single-lead sinus-rhythm ECG + 6 EHR-derived clinical risk factors	Concurrent/near-term AF (within 31 days)	CNN (ResNet) on lead-I ECG fused with a Random Forest classifier for the risk factors	AUC 0.76 in an age/sex-matched dataset, rising to 0.88 in an unmatched replication dataset; performance matched that of a 12-lead ECG AI model
Gadaleta et al. 2023 [18]	459,889 two-week chest-patch recordings	AF-free single-lead (modified lead II) ECG, 10-min–24-h windows	“Near-term” AF within a 14-day patch period (any AF during recording)	Ensemble DL using morphology, HRV, ectopy and feature-based models	AUC of 0.77 for 10-min input and of 0.80 for 24 h input for 14-day AF prediction; outputs calibrated using isotonic regression, but no calibration metrics are reported
Hygrell et al. 2023 [35]	Safer & STROKESTOP-style handheld ECG cohorts	Single-lead sinus-rhythm ECG (handheld recordings)	Paroxysmal AF over repeated handheld ECG recordings	CNN applied to ECG images/signals	AUC ≈ 0.80 in age-diverse SAFER cohort; ≈0.62 in older, age-homogeneous cohort
Jabbour et al. 2024 [36]	Tertiary hospital, retrospective cohort with ECG, clinical data and genetics	12-lead ECG + clinical variables + polygenic risk	Incident AF over the years	DL-ECG model + clinical risk model + polygenic score	ECG-AI AUC ≈ 0.78; adding CHARGE-AF and polygenic score modestly improved model fit with minimal AUC change but better reclassification; ECI of 0.086; DCA reported
Khurshid et al. 2022 [30]	Health-system retrospective ECG database	12-lead ECG + clinical covariates	Incident AF over follow-up	DL-ECG AF-risk score plus clinical variables	ECG-AI alone outperformed CHARGE-AF; combined ECG-AI + clinical model yielded modest additional gain; ICI of 0.0035–0.0212; improved NRI reported
Kim et al. 2022 [37]	Retrospective cohort of 1166 pts with 24 h Holter (sinus-rhythm segments)	Multi-lead 24-h Holter ECG	History of paroxysmal AF (present vs. absent in Holter) inferred from sinus-rhythm data	DL model on sinus-rhythm Holter segments	AUC ≈ 0.84–0.85; slightly higher performance for night-time segments
Lee et al. 2025 [29]	Retrospective cohort of 121,600 Korean pts (development) + CODE 15% (external validation)	Standard 12-lead sinus-rhythm ECG images	Concurrent paroxysmal or incident AF within 2 years	Modified CNN	Internal AUC 0.907; external interethnic validation AUC of 0.884, increasing to 0.906 after adjustment for age and sex
Melzi et al. 2023 [38]	Longitudinal ECG retrospective cohorts with repeated tracings	12-lead sinus-rhythm ECG with time-interval and longitudinal features	New-onset AF with explicit use of longitudinal ECG information	DNNs with time-interval and longitudinal modules	Incremental value of longitudinal ECG information over single-time-point models (higher discrimination and reclassification metrics)
Noseworthy et al. 2022 [31]	Pragmatic non-randomized trial on a prospective cohort of 1003 pts	Routine 12-lead sinus-rhythm ECG + 30-day patch	AF detection during up to 30 days; AF diagnosis vs. matched usual care	AI-ECG AF-risk to select pts for patch	AF detected in 7.6% AI-high-risk vs. 1.6% AI-low-risk; AF diagnosis ≈ 10.6% vs. 3.6% vs. usual care; HR ≈ 2.85; no stroke/mortality endpoints
Rabinstein et al. 2021 [39]	Retrospective cohort of 930 stroke pts (ESUS vs. known mechanism)	In-hospital ECGs + post-discharge ambulatory monitoring	AF detection on ambulatory monitoring after ESUS	Application of Attia AI-ECG probability to index ECGs	AI-ECG probability > 0.20 associated with OR ≈ 5.47 for AF detected on prolonged monitoring in ESUS pts
Raghunath et al. 2021 [15]	430,909 pts, 1.6 M ECGs, no prior AF (retrospective cohort)	12-lead sinus-rhythm ECG	New-onset AF within 1 year and AF-related stroke up to 30 years	Large-scale DNN on ECG	1-year AF AUC of 0.85; HR ≈ 7.2 for high- vs. low-risk over 30 years; number needed to screen ≈ 9 to find one new AF case
Sau et al. 2025 [40]	Large, multi-center, retrospective clinical datasets	ECG + clinical data	Incident AF; comparison of AI vs. clinical risk scores	Multiple ML/DL models vs. CHARGE-AF and others	AI-ECG and EHR-based models generally outperformed traditional scores; combined models provided the best discrimination; Brier scores were 0.089–0.107; and positive NRI was reported when combined with clinical scores
Schoels et al. 2025 [41]	Retrospective cohort of stroke-unit pts	ECGs, continuous telemetry, clinical variables	Incident AF during and after stroke-unit admission	ML/DL models integrating ECG and clinical data	Improved AF detection vs. rule-based strategies; validation AUCs ≈ 0.80 reported
Singh et al. 2022 [19]	24-h Holter retrospective recordings without AF at baseline	24-h ambulatory ECG	AF occurrence within 15 days after AF-free Holter	Deep-learning meta-model combining HR trend, beat-level features, and PAC counts	External-validation AUC of 0.76 for 15-day AF prediction
Wu et al. 2024 [42]	Clinical ECG device (retrospective cohort)	10-s 12-lead ECG converted to images	Undetected AF or AF within the past 2 years	Lightweight 4-block CNN	AUC comparable to server-side (0.82 for internal, 0.80 for external validation)
Yuan et al. 2023 [43]	US Veterans retrospective cohort	12-lead sinus-rhythm ECG	Presence of AF within 31 days of the sinus-rhythm ECG (secondary outcome: incident AF over 1 year)	DL-ECG model on raw ECG	Reported c-statistics ≈ 0.86–0.93; AI-ECG outperformed traditional clinical risk factors alone; good calibration was evaluated using Spiegelhalter z-test; Brier score of 0.02
Zeidaabadi et al. 2025 [27]	Health-system ECG image retrospective cohort	Rasterised 12-lead ECG images (PDF/scans)	Incident AF prediction	Image-based CNN; combination with clinical risk scores	C-statistics ≈ 0.72–0.75 for image-AI alone; adding clinical scores improved net reclassification; Brier score of 0.072–0.149; improved NRI (0.378) when combined with clinical scores
Short-term AF onset					
Boon et al. 2016 [44]	PhysioNet AFPDB (highly curated case–control dataset)	HRV segments shorter than 30 min (e.g., 5-min)	PAF onset vs. control; evaluation of reduced-length segments (minutes)	Baseline HRV feature + SVM system	Achieved 79.3% prediction accuracy using 15-min HRV segments and 68.9% using 10-min segments
Boon et al. 2018 [45]	PhysioNet AFPDB (highly curated case–control dataset)	5-min HRV segments (reduced from typical 30-min)	PAF vs. non-PAF HRV segments; imminent PAF prediction (minutes)	Non-dominated Sorting Genetic Algorithm III to optimize HRV feature extraction + SVM classifier	Overall accuracy of 87.7%; sensitivity can be increased at the expense of specificity (explicit trade-off discussed)
Castro et al. 2021 [46]	PhysioNet AFPDB (highly curated case–control dataset)	2- and 5-min HRV windows	PAF vs. non-PAF; evaluation of short HRV windows (2–5 min)	Recursive feature elimination + ML classifiers	Precision of 93.24% with a 5-min window and 89.21% with 2-min window (10-fold cross-validation)
Ebrahimzadeh et al. 2018 [47]	PhysioNet AFPDB (highly curated case–control dataset)	≈30-min HRV segments	PAF vs. non-PAF (imminent AF prediction, ≈30 min)	Large combined HRV feature vector + mixture-of-experts classifier	Overall accuracy of 98.21%, exceeding classical ML baselines (≈91.9–93.8%)
Grégoire et al. 2025 [48]	Retrospective Holter database (95,871 recordings; 1319 PAF episodes)	24-h Holter; HRV windows ≥5 min in normal sinus rhythm	AF episode within the next ≈1 day; mean prediction lead ≈15 h	Gradient-boosted decision tree using HRV and heart-rate fragmentation features	AUC of 0.919 (95% CI 0.879–0.958) and AUPRC of 0.919 for ≥5-min SR windows preceding AF onset
Mohebbi and Ghassemian 2012 [49]	PhysioNet AFPDB (highly curated case–control dataset)	30-min RR-interval/HRV windows	PAF vs. non-PAF segments; prediction up to ≈30–45 min ahead	Spectrum and bispectrum features + SVM classifier	Sensitivity of 96.30%, specificity of 93.10%, and positive predictivity of 92.86%
Narin et al. 2018 [21]	PhysioNet AFPDB (highly curated case–control dataset)	5-min HRV segments from RR-intervals	PAF vs. control segments; prediction 2.5–7.5 min before PAF onset	Time- and frequency-domain HRV features + GA-based feature selection + k-NN classifier	Sensitivity of 92%, specificity of 88%, and accuracy of 90% for 2.5–7.5-min pre-PAF interval
Rooney et al. 2023 [20]	PhysioNet Retrospective continuous ECG recordings, with AF episodes	2-lead or multi-lead ECG segments from 24-h recordings	AF onset forecasting 7.5–60 min ahead from pre-AF sinus rhythm	CNN + transformer architectures on long-term ECG windows	AUC ≈ 0.74 at 7.5-min lead; risk trajectories diverged ≈ 15 min before AF onset

Abbreviations: AF, atrial fibrillation; AI, artificial intelligence; AUC, area under the curve (receiver operating characteristic); AUPRC, area under the precision–recall curve; CI, confidence interval; CNN, convolutional neural network; DCA, decision curve analysis; DL, deep learning; DNN, deep neural network; ECG, electrocardiogram; ECI, estimated calibration index; EHR, electronic health record; ESUS, embolic stroke of undetermined source; Fed-CL, federated convolutional neural network–long short-term memory; GA, genetic algorithm; HR, hazard ratio/heart rate; HRV, heart rate variability; ICI, integrated calibration index; k-NN, k-nearest neighbors; LSTM, long short-term memory; ML, machine learning; NRI, net reclassification improvement; SR, sinus rhythm; OR, odds ratio; PAC, premature atrial contraction; PAF, paroxysmal atrial fibrillation; pts, patients; RNN, recurrent neural network; RR, R-R interval; SVM, support vector machine; UK, United Kingdom; US, United States.

## 4. AI Applied to Ambulatory Holter and Patch Monitoring

### 4.1. Hidden AF in 24-h Holter Recordings

Several recent studies have focused on AI analysis of 24-h Holter recordings to detect “hidden” AF in patients whose analyzed segments are entirely in sinus rhythm. Kim and colleagues developed a model that processes entire Holter recordings and predicts the presence of paroxysmal AF even when AF episodes are excluded from the input. In a cohort of 1166 individuals, their algorithm achieved AUCs around 0.85 and slightly higher performance for night-time segments, consistent with a more stable sinus rhythm and autonomic influence overnight [37].

Chang et al. built a two-stage deep-learning architecture using short-time Fourier transform spectrograms from three Holter leads (simulated V_1_, V_5_, II) during sinus rhythm. A contemporary convolutional architecture (ConvNeXt), designed to simulate Transformer micro-characteristics, first classified 60-s segments as high- or low-risk. A second-stage Long Short-Term Memory (LSTM) aggregated ten consecutive segments (10-min window) to produce patient-level predictions. Segment-level performance was characterized by high sensitivity (0.91) but moderate specificity (0.57), while patient-level predictions achieved an AUC of 0.87, accuracy of 0.82, and balanced sensitivity and specificity around 0.83 and 0.82, respectively, with slightly superior metrics overnight. In comparison, within the same cohort, traditional risk scores, including Taiwan AF, C_2_HEST, and HATCH, had AUCs between 0.72 and 0.79, inferior to the AI model [50].

These Holter-based studies highlight that subtle, temporally varying features within sinus rhythm, such as beat-to-beat variability, ectopy patterns, and spectral content, encode information about the underlying arrhythmogenic substrate that is not captured by static clinical scores. They also illustrate the importance of aggregating information across time to reduce noise and false positives.

### 4.2. Near-Term AF Prediction from Ambulatory Monitoring

Moving beyond the classification of “ever AF” within a recording, several groups have attempted to forecast AF over near-term horizons using Holter or patch recordings without overt AF. Singh et al. trained a deep neural network on 24-h AF-free ambulatory ECGs to predict AF occurrence within 15 days, using a meta-model that ingests heart-rate trends, beat-level information, and premature atrial contraction counts. In external validation, the ensemble achieved an AUC of 0.76 for 15-day AF prediction [19]. Gadaleta et al. analyzed at-home single-lead chest-patch data from more than 450,000 two-week recordings; they deliberately restricted inputs to AF-free segments (10 min to 24 h) and trained an ensemble of deep learning (DL) and feature-based models to predict AF within the 14-day patch period. Their best model achieved an AUC of 0.77 for 10-min inputs and 0.80 for 24-h inputs, with calibration adequate across age and risk strata and somewhat lower discrimination in very elderly, homogeneous cohorts [18]. Rooney and colleagues focused specifically on imminent AF forecasting in long-term ECG, applying CNN-transformer architectures to 24-h recordings to predict AF onset from 7.5 to 60 min ahead. They reported AUCs around 0.74 at a 7.5-min lead time, with divergence in predicted risk curves appearing approximately 15 min before AF onset, suggesting a gradual transition toward an unstable electrophysiologic state [20]. Together, these ambulatory studies bridge the gap between purely substrate-oriented predictions (horizon within years) and beat-to-beat, “impending” arrhythmia detection, enabling new, risk-adaptive monitoring strategies and numerous applications, including wearable-based, on-device models to trigger intensified sampling or alerts when short-term AF risk spikes (Table 1).

## 5. HRV-Based Short-Term Prediction of Paroxysmal AF

Decades of work on HRV preceded modern deep learning, yet it remains relevant, especially for resource-constrained or edge-device applications where full waveform DL may be infeasible. Early studies using the PhysioNet Atrial Fibrillation Prediction Database showed that PAF can be anticipated several minutes in advance based on HRV features. Narin and colleagues reported that 5-min HRV segments 2.5–7.5 min before PAF onset could be distinguished from control segments with a sensitivity of 92%, a specificity of 88%, and an accuracy of 90% using carefully selected time- and frequency-domain features and k-nearest neighbor classification [21]. Mohebbi and Ghassemian’s analysis of 30-min RR-interval windows achieved a sensitivity of 96% and a specificity of 93% for predicting imminent AF episodes by distinguishing them from periods of normal sinus rhythm distant from an event, although on relatively small and well-annotated datasets [49]. Interestingly, a comparative study by Grégoire et al. demonstrated that decision tree models based on handcrafted HRV features from 5-min recordings can outperform deep learning approaches trained on raw, 30-s ECG signals, identifying short-term vagal activity and heart rate fragmentation as the most significant predictors of imminent atrial fibrillation [48].

Similar works incorporated further nonlinear features to optimize feature subsets and classifier operating points. Ebrahimzadeh and colleagues combined a large set of HRV descriptors with a mixture-of-experts classifier, reporting an overall accuracy of 98.2% for AF onset prediction, again in the same PhysioNet cohort [47]. This aligns with the paradigms of Schoels et al. and Singh et al., which deploy ensemble meta-models that fuse extracted HRV parameters, beat-level morphologic features, and other tabular data [19,41]. This approach highlights the broader utility of ensemble and hybrid modeling strategies. While contemporary deep learning excels at processing unstructured, raw ECG waveforms directly, ensemble learning achieves a consensus or “jury” effect by aggregating the outputs of multiple base algorithms. However, classical ensemble methods, such as bagging or gradient boosting, are effective when applied to structured, hand-crafted clinical variables, which is rarely the case in contemporary deep learning approaches employing raw signals. Finally, another study has updated these approaches using modern feature-selection algorithms and machine learning classifiers, demonstrating an accuracy of 88% for predicting AF events using HRV windows as short as five minutes [45].

While impressive, these HRV-based results must be interpreted cautiously: appraising the performance is difficult, since these studies present such diverse designs that make them hardly comparable. For example, studies utilizing the widely overused PhysioNet AF Prediction Database report extraordinary accuracies between 90% and 98% [21,47,49]. However, these figures are often derived from highly curated case–control cohorts utilizing non-pragmatic splits. These artificially inflated metrics cannot be directly compared to population-scale prospective cohort studies, where the true AF incidence is vastly lower, and the natural class imbalance drastically reduces real-world positive predictive value. Nevertheless, they confirm an important proof of concept that pre-AF dynamics exist and are detectable even from RR-interval patterns alone, supporting the plausibility of deep-learning approaches that integrate HRV implicitly.

## 6. Conventional Risk Scores vs. AI-Based Models

Before AI-based models, risk stratification for incident AF relied on multivariable scores derived from epidemiologic cohorts. The CHARGE-AF model incorporates the following demographic and clinical variables: age, race, height, weight, blood pressure, smoking, antihypertensive therapy, heart failure, diabetes, and myocardial infarction [8] to predict 5-year AF risk [51]. Notably, the inclusion of race as a variable in traditional clinical scores like CHARGE-AF would benefit from additional nuance when compared to AI models. Given the ongoing debate regarding race as a social rather than strictly biological construct, its use in predictive modeling raises first-order concerns regarding algorithmic fairness and the potential to perpetuate health disparities. This poses a challenge that modern AI-ECG models must also rigorously audit across diverse, multi-ethnic cohorts to ensure equitable care delivery. Similarly, the C_2_HEST score (coronary artery disease or chronic obstructive pulmonary disease, hypertension, elderly, systolic heart failure, thyroid disease) was developed in large Chinese and Korean cohorts as a simple tool to predict incident AF [4], and a modified mC_2_HEST score incorporating finer age stratification was recently validated [52]. Complementary work from Taiwan used nationwide administrative data on more than 7.2 million adults to derive a Taiwan AF score [6], while the HATCH score, originally designed to predict AF progression [7], has also been validated as a tool to predict new-onset AF [5].

Across the short-term and long-term domains, a consistent theme is that AI-based ECG and HRV models outperform clinical risk scores when both are tested in the same cohorts. In Chang’s Holter study, patient-level AI models achieved AUC ≈ 0.87 compared with 0.72 to 0.79 for Taiwan AF, C_2_HEST, mC_2_HEST, CHA_2_DS_2_-VASc, and HATCH [50]. In Gadaleta’s chest-patch study, AI-derived patch risk generally exceeded the discrimination of the baseline age- and gender-based clinical model for near-term AF detection, particularly in younger, more heterogeneous populations [18]. Similarly, a study by Yuan et al. showed that a CNN predicted the presence of AF within 31 days with AUC values of 0.86 and 0.93 for two cohorts. The DL model’s performance remained consistent across racial and demographic subgroups and outperformed traditional clinical risk factors like the CHA_2_DS_2_-VASc score [43]. In incident AF prediction, AI-ECG models from Attia, Raghunath, and others show c-statistics in the 0.78–0.85 range, which are comparable to CHARGE-AF and C_2_HEST scores in external validations. Jabbour’s study integrating DL-ECG, clinical models, and polygenic scores found that the ECG-AI score alone performed comparably to or better than CHARGE-AF, while the addition of clinical information modestly improved risk stratification [36].

While AI models frequently report comparable performance to clinical scores, such comparisons can sometimes be superficial. A methodologically fair comparison requires testing both modalities head-to-head on the exact same patient cohorts, with identical follow-up windows and outcome definitions [36,40]. Unfortunately, this rigorous head-to-head evaluation is not done frequently in the literature and is particularly vulnerable to bias in retrospective datasets due to mismatched follow-up durations and differing baseline ascertainment methods. Consequently, these risk stratification tools should currently be viewed as complementary rather than purely competitive.

Notably, while established metrics exist for latent and long-term AF risk, no clinical risk scores or other indicators exist for predicting imminent AF, aside from the more traditional HRV and recent DL solutions.

## 7. Current Gaps, Clinical Utility, and Future Directions

Most AI-based AF models are developed using retrospective data from isolated health systems or specific ECG vendors. Even when datasets are large, they often fail to reflect the true diversity of real-world populations, leading to site-specific training constraints where models learn local institutional artifacts rather than universal physiological signals. Furthermore, a significant risk of bias is often found and must be carefully weighed when interpreting reported performance metrics. For example, several models trained on highly curated, open datasets, such as the PhysioNet AF Prediction Database, report accuracies between 90% and 98% [21,47,49]. However, these figures often present with spectrum bias and class imbalance mishandling, and, naturally, they cannot directly be compared to population-scale prospective cohorts where true AF prevalence is vastly lower. Additionally, models utilizing 24-h Holter and patch monitoring face inherent risks of label leakage if the precise temporal boundary between the sinus-rhythm input window and the AF event is not rigorously isolated.

Systematic evaluations of algorithmic fairness also remain underdeveloped, since the field of predicting hidden AF from sinus rhythm is still relatively “young” compared to overt AF detection. While initial inter-ethnic validations are emerging [29], systematic discussions of model performance across diverse subgroups are still lacking. Deep learning algorithms do possess the potential to inadvertently exert biases inherently present in their training data, and, consequently, mandatory reporting of subgroup performance and fairness audits must become standard practice to ensure these models do not exacerbate existing health disparities [53]. Open science, specifically by sharing data and models, is a related concern and potential solution. Transparent reporting of dataset composition, labeling procedures, and preprocessing is essential for clinicians to assess generalizability and ensure fairness. Systematic application of established standards, such as TRIPOD + AI and others [53,54], along with the deposition of models into standardized repositories, like PhysioNet (https://physionet.org) or GitHub (https://github.com), is necessary to move beyond closed silos and into reproducible clinical practice.

Additionally, for successful clinical deployment, calibration (the agreement between predicted and observed risk) and net clinical benefit are arguably as important as discrimination. Following the TRIPOD + AI guidelines, both discrimination and calibration should be standard reporting requirements [53]. Yet, as seen in Table 1, our analysis reveals that fewer than 20% of the evaluated AI-AF studies reported calibration metrics (e.g., Brier score, Integrated Calibration Index [ICI], or other calibration indices), net reclassification improvement (NRI), or formal decision-curve analyses [18,23,27,30,36,40,43]. Because discrimination is more frequently reported, it remains the most actionable metric currently available for comparison. However, the systematic omission of calibration metrics undermines pragmatic integration, as poorly calibrated models may severely overestimate or underestimate risk in specific subpopulations, limiting their safe translation into shared decision-making. To provide a calibrated sense of trustworthiness regarding these methodological limitations, a formal PROBAST + AI-based risk-of-bias assessment for each included study is provided in Supplementary Materials [55].

Another issue to be addressed is deep neural network interpretability, as CNNs and transformers are often “black boxes”, raising concerns about clinical trust and acceptance [53]. To address this, recent studies integrate Explainable AI (XAI) techniques, such as Gradient-weighted Class Activation Mapping (Grad-CAM) or other saliency maps. These visual tools reveal that incident AF prediction models typically focus on P-wave morphology, the PR interval, or other subtle features [27,30]. However, XAI methods are approximate. Evaluations demonstrate that algorithmic attention can be misdirected, exploiting baseline noise or misinterpreting features [56]. Thus, while XAI improves transparency, visual explanations require rigorous clinical validation, and future models may benefit from hybrid approaches incorporating explicit atrial biomarkers. Such models, combining explicit traits, like P-wave indices, echocardiographic strain, and MRI fibrosis, along with learned representations, may help bridge mechanistic and predictive domains [13,40].

Finally, overcoming the barriers related to accurate and reliable AF prediction raises one final concern: how clinically relevant can such outcomes become, and what are the therapeutic options thereafter? To date, the vast majority of AI-AF studies treat AF detection as the primary endpoint. However, subclinical AF and short AF runs have variable and sometimes modest associations with stroke; SCAF episodes ≥24 h carry the highest risk, while shorter episodes are less clearly actionable, especially when they are asymptomatic. Broad screening for subclinical AF in the general population has yielded debated results regarding the net clinical benefit of anticoagulation, as highlighted by the LOOP and STROKESTOP II trials [57,58]. This ambiguity is heavily reinforced by recent randomized trials of device-detected subclinical AF, such as NOAH-AFNET 6 and ARTESiA, which demonstrated that anticoagulating short subclinical AF episodes does not uniformly reduce stroke without a concomitant increase in major bleeding [59,60]. If AI-guided strategies mainly increase detection of very low-burden AF, they may yield little net benefit while exposing patients to bleeding risks from anticoagulation and anxiety from “arrhythmia labeling”, and this is a new spectrum of individuals, not necessarily patients, to be explored [61,62]. Consequently, AI algorithms may hold the greatest practical value only when applied to subpopulations with an inherently high risk of stroke or hemodynamic compromise. Such a subpopulation could be patients with embolic stroke of undetermined source (ESUS), where AI-ECG models could identify those with a high probability of harboring an arrhythmogenic substrate, justifying the allocation of prolonged continuous monitoring resources to capture actionable AF and transition patients to oral anticoagulation [39]. In further vulnerable populations, especially those with heart failure, incident AF frequently triggers hemodynamic decompensation, and early rhythm control improves outcomes, as shown by the EAST-AFNET 4 trial [63,64]. Predicting AF onset in these high-risk scenarios could enable proactive surveillance and the timely implementation of an early rhythm-control strategy. Therefore, the immediate clinical integration of AI-ECG should focus on these targeted populations, shifting care from reactive treatment to proactive, personalized prevention. Prospective randomized trials must therefore define composite outcomes that include stroke, systemic embolism, heart failure hospitalization, cognitive decline, and quality of life, but also determine the subpopulations that could benefit from this strategy. The ASSERT and related device-based studies provide a foundation for burden-risk relationships but were not designed around AI-guided prediction [61,65].

From a broader evaluation standpoint, health economics and cost-effectiveness analyses of AI-guided sinus-rhythm screening are currently largely absent from the literature, as the clinical benefit of identifying latent AF is still being investigated. However, for future clinical implementation, the costs associated with deploying AI infrastructure and the subsequent downstream testing should be offset by savings from possible stroke reduction and proactive care [66]. Furthermore, from a regulatory standpoint, while most of these predictive models remain investigational, the governance of these tools is already shifting. The recently issued EU AI Act represents a paradigm shift in standard establishment, strictly classifying medical AI as high-risk and guiding its development and use [67]. Future predictive AI-ECG approaches will need to comply with regulations regarding data privacy, risk management, and human oversight before they can transition to routine clinical tools.

## 8. Conclusions

AI is changing how AF risk is understood, shifting the focus from an intermittently appearing rhythm problem to an underlying electrical and structural atrial phenotype that exists before AF is clinically apparent and can be inferred from sinus-rhythm ECG recordings across different time scales. Across modalities, deep-learning models applied to 12-lead ECGs, ECG images, Holter and patch data, and single-lead wearables generally outperform, or are at least comparable to, traditional clinical risk scores for detecting latent AF and predicting long-term incident AF, while HRV-based approaches show that imminent AF is often preceded by short-term changes in autonomic tone and beat-to-beat variability.

At the same time, AI-based AF prediction should currently be viewed as a supplement rather than a replacement for guideline-directed care [32]. While some models do offer superior, or at least comparable, risk stratification, their current clinical role is to augment human decision-making by identifying high-risk individuals who warrant targeted monitoring, rather than overriding standard diagnostic and therapeutic pathways. Furthermore, most models remain retrospective, come from one or a few centers, and have limited external validation and variable calibration. Only a single pragmatic trial has tested AI-guided screening in routine practice, and a reduction in stroke or mortality has not been shown yet. Future work should therefore emphasize multicenter randomized or pragmatic trials with hard clinical endpoints, robust external validation, and shared benchmarks and implementation strategies that explicitly address calibration and transparency. If these challenges are met, AI-enabled ECG and ambulatory monitoring could support a shift from opportunistic, reactive AF detection to more proactive, substrate- and burden-guided care, with the potential to improve prevention of stroke and other adverse cardiovascular events.

## Data Availability

No new data were created or analyzed in this study.

## Supplementary Materials

The following supporting information can be downloaded at: https://www.mdpi.com/article/10.3390/biomedicines14051058/s1, A. Preferred Reporting Items for Systematic reviews and Meta-Analyses extension for Scoping Reviews (PRISMA-ScR) Checklist; B. Search Strategy and Results; C. PROBAST+AI Assessment of Included Studies.
