# Supplementary material for: Artificial Intelligence for Prediction and Detection of Atrial Fibrillation from Sinus-Rhythm Electrocardiograms and Ambulatory Monitoring
Source: Biomedicines. 2026 May 7;14(5):1058. doi: 10.3390/biomedicines14051058 (PMC13204813; doi:10.3390/biomedicines14051058)
Supplement: Supplementary file 1 [file biomedicines-14-01058-s001.zip › biomedicines-4298205-supplementary.pdf]

A. Preferred Reporting Items for Systematic reviews and Meta-Analyses extension for Scoping Reviews (PRISMA-ScR [1]) Checklist

SECTION	ITEM	PRISMA-ScR CHECKLIST ITEM	REPORTED ON PAGE #
**TITLE**			
Title	1	Identify the report as a scoping review.	1
**ABSTRACT**			
Structured summary	2	Provide a structured summary that includes (as applicable): background, objectives, eligibility criteria, sources of evidence, charting methods, results, and conclusions that relate to the review questions and objectives.	1
**INTRODUCTION**			
Rationale	3	Describe the rationale for the review in the context of what is already known. Explain why the review questions/objectives lend themselves to a scoping review approach.	2
Objectives	4	Provide an explicit statement of the questions and objectives being addressed with reference to their key elements (e.g., population or participants, concepts, and context) or other relevant key elements used to conceptualize the review questions and/or objectives.	2
**METHODS**			
Protocol and registration	5	Indicate whether a review protocol exists; state if and where it can be accessed (e.g., a Web address); and if available, provide registration information, including the registration number.	2, 3
Eligibility criteria	6	Specify characteristics of the sources of evidence used as eligibility criteria (e.g., years considered, language, and publication status), and provide a rationale.	3, Suppl. Material
Information sources*	7	Describe all information sources in the search (e.g., databases with dates of coverage and contact with authors to identify additional sources), as well as the date the most recent search was executed.	3, Suppl. Material
Search	8	Present the full electronic search strategy for at least 1 database, including any limits used, such that it could be repeated.	3, Suppl. Material
Selection of sources of evidence†	9	State the process for selecting sources of evidence (i.e., screening and eligibility) included in the scoping review.	3, Suppl. Material
Data charting process‡	10	Describe the methods of charting data from the included sources of evidence (e.g., calibrated forms or forms that have been tested by the team before their use, and whether data charting was done independently or in duplicate) and any processes for obtaining and confirming data from investigators.	3, Suppl. Material
Data items	11	List and define all variables for which data were sought and any assumptions and simplifications made.	3, Suppl. Material and throughout main text

SECTION	ITEM	PRISMA-ScR CHECKLIST ITEM	REPORTED ON PAGE #
Critical appraisal of individual sources of evidence§	12	If done, provide a rationale for conducting a critical appraisal of included sources of evidence; describe the methods used and how this information was used in any data synthesis (if appropriate).	-
Synthesis of results	13	Describe the methods of handling and summarizing the data that were charted.	Throughout main text
**RESULTS**			
Selection of sources of evidence	14	Give numbers of sources of evidence screened, assessed for eligibility, and included in the review, with reasons for exclusions at each stage, ideally using a flow diagram.	3-12
Characteristics of sources of evidence	15	For each source of evidence, present characteristics for which data were charted and provide the citations.	3-12
Critical appraisal within sources of evidence	16	If done, present data on critical appraisal of included sources of evidence (see item 12).	3-12, Suppl. Material
Results of individual sources of evidence	17	For each included source of evidence, present the relevant data that were charted that relate to the review questions and objectives.	3-12, Suppl. Material
Synthesis of results	18	Summarize and/or present the charting results as they relate to the review questions and objectives.	3-12
**DISCUSSION**			
Summary of evidence	19	Summarize the main results (including an overview of concepts, themes, and types of evidence available), link to the review questions and objectives, and consider the relevance to key groups.	12-15
Limitations	20	Discuss the limitations of the scoping review process.	12-14
Conclusions	21	Provide a general interpretation of the results with respect to the review questions and objectives, as well as potential implications and/or next steps.	15
**FUNDING**			
Funding	22	Describe sources of funding for the included sources of evidence, as well as sources of funding for the scoping review. Describe the role of the funders of the scoping review.	15

Notes. JBI = Joanna Briggs Institute; PRISMA-ScR = Preferred Reporting Items for Systematic reviews and Meta-Analyses extension for Scoping Reviews.

* Where sources of evidence (see second footnote) are compiled from, such as bibliographic databases, social media platforms, and Web sites.

† A more inclusive/heterogeneous term used to account for the different types of evidence or data sources (e.g., quantitative and/or qualitative research, expert opinion, and policy documents) that may be eligible in a scoping review as opposed to only studies. This is not to be confused with information sources (see first footnote).

‡ The frameworks by Arksey and O'Malley (6) and Levac and colleagues (7) and the JBI guidance (4, 5) refer to the process of data extraction in a scoping review as data charting.

§ The process of systematically examining research evidence to assess its validity, results, and relevance before using it to inform a decision. This term is used for items 12 and 19 instead of "risk of bias" (which is more applicable to systematic reviews of interventions) to include and acknowledge the various sources of evidence that may be used in a scoping review (e.g., quantitative and/or qualitative research, expert opinion, and policy document).

B. Search Strategy and Results

Electronic Database Search String

The literature search was conducted across Medline/OVID, Embase/OVID and Scopus. The following search string represents the core strategy applied:

```
("artificial intelligence" OR "deep learning" OR "machine learning") AND ("atrial fibrillation") AND ("electrocardiogra*") AND ("sinus" OR "SR" OR "normal") AND "prediction"
```

Screening and Selection Results

- Initial Yield: 459 titles identified through electronic database searches and citation snowballing.
- Deduplication & Screening: Following the removal of duplicates, title and abstract screening excluded non-relevant articles.
- Full-Text Assessment: Full-text articles were independently assessed by two reviewers for eligibility. Exclusions at this stage primarily involved non-peer-reviewed preprints, conference abstracts and models designed to detect concurrent AF from AF-containing ECGs rather than predicting/detecting it from sinus rhythm. In case of discrepancy a third reviewer joined and the decision was based on majority voting.
- Final Inclusion: 32 peer-reviewed original research articles were finally included.

C. PROBAST+AI Assessment of Included Studies

Study	D1: Participants & data sources	D2: Predictors	D3: Outcomes	D4: Analysis	Overall RoB	Overall applicability concern	Key remarks
Long-term onset or latent AF							
Alreshidi et al. 2024 *[2]*	RoB: (+) App: (+)	RoB: (+) App: (+)	RoB: (+) App: (+)	RoB: (+)	Low	Low	Multi-institution dataset utilizing federated learning to preserve data privacy.
Attia et al. 2019 *[3]*	RoB: (?) App: (+)	RoB: (+) App: (+)	RoB: (?) App: (+)	RoB: (?)	Unclear	Low	Retrospective single-center design (Mayo Clinic). Possible outcome conflation. No prospective external validation within the study.
Baek et al. 2021 *[4]*	RoB: (-) App: (-)	RoB: (+) App: (+)	RoB: (-) App: (?)	RoB: (?)	High	High	Single-center hospital cohort (Inha University). High risk of spectrum bias and lack of external validation.
Brant et al. 2025 *[5]*	RoB: (+) App: (+)	RoB: (+) App: (+)	RoB: (+) App: (+)	RoB: (+)	Low	Low	Multinational community cohorts (Framingham, UK Biobank, ELSA-Brasil). Excellent generalizability and strict temporal outcome definitions.
Cho et al. 2025 *[6]*	RoB: (+) App: (+)	RoB: (+) App: (+)	RoB: (+) App: (+)	RoB: (+)	Low	Low	Extensive external validations across multinational cohorts (Severance, UK Biobank, Mayo, PTB-XL). Low risk of bias.
Choi et al. 2024 *[7]*	RoB: (+) App: (+)	RoB: (+) App: (+)	RoB: (+) App: (+)	RoB: (-)	High	Low	Utilized serial ECGs. External validation performed at an independent hospital (Wonju Severance), yet age dominated over ECG predictors.
Christopoulos et al. 2020 *[8]*	RoB: (-) App: (+)	RoB: (+) App: (+)	RoB: (+) App: (+)	RoB: (+)	Unclear	Low	Good long-term incident AF tracking, but relies on a single geographical region (Olmsted County/Mayo Clinic).
Dupulthys et al. 2023 *[9]*	RoB: (+) App: (+)	RoB: (+) App: (+)	RoB: (-) App: (+)	RoB: (+)	Unclear	Low	Evaluated single-lead + clinical factors but outcome time horizons varied (within 31 days) creating some ambiguity between true prediction vs. delayed diagnosis. Retrospective single-center.

Gadaleta et al. 2023 *[10]*	RoB: (+) App: (+)	RoB: (+) App: (+)	RoB: (+) App: (+)	RoB: (+)	Low	Low	Uses 24-h ambulatory patch data. Rigorous isolation of sinus-rhythm inputs to prevent label leakage before the 14-day AF prediction window.
Hygrell et al. 2023 *[11]*	RoB: (+) App: (+)	RoB: (+) App: (+)	RoB: (+) App: (+)	RoB: (+)	Low	Low	Externally validated across distinct screening cohorts (SAFER, STROKESTOP).
Jabbour et al. 2024 *[12]*	RoB: (+) App: (+)	RoB: (+) App: (+)	RoB: (+) App: (+)	RoB: (+)	Low	Low	Good temporal separation for incident AF; external validation successfully performed on the MIMIC-IV dataset.
Khurshid et al. 2022 *[13]*	RoB: (+) App: (+)	RoB: (+) App: (+)	RoB: (+) App: (+)	RoB: (+)	Low	Low	Multi-hospital training (MGH) and independent external testing sets (BWH, UK Biobank). Proper temporal separation.
Kim et al. 2022 *[14]*	RoB: (-) App: (-)	RoB: (+) App: (+)	RoB: (-) App: (?)	RoB: (?)	High	High	Single-center retrospective Holter data; lacks external validation.
Lee et al. 2025 *[15]*	RoB: (+) App: (+)	RoB: (+) App: (+)	RoB: (+) App: (+)	RoB: (+)	Low	Low	Large Korean development set with explicit interethnic external validation (CODE dataset).
Melzi et al. 2023 *[16]*	RoB: (-) App: (-)	RoB: (+) App: (+)	RoB: (-) App: (?)	RoB: (?)	High	High	Single-center data (La Princesa Hospital). No external validation.
Noseworthy et al. 2022 *[17]*	RoB: (+) App: (+)	RoB: (+) App: (+)	RoB: (+) App: (+)	RoB: (+)	Low	Low	Pragmatic, prospective, non-randomized design assessing real-world targeted screening vs. usual care. High clinical applicability.
Rabinstein et al. 2021 *[18]*	RoB: (-) App: (-)	RoB: (+) App: (+)	RoB: (+) App: (+)	RoB: (?)	High	High	Retrospective, single-center ESUS population limits generalizability.
Raghunath et al. 2021 *[19]*	RoB: (-) App: (-)	RoB: (+) App: (+)	RoB: (+) App: (+)	RoB: (+)	High	High	Excellent long-term outcome tracking, but completely reliant on a single regional health system (Geisinger). High concern for lack of multi-ethnic generalizability.

Sau et al. 2025 *[20]*	RoB: (+) App: (+)	RoB: (+) App: (+)	RoB: (+) App: (+)	RoB: (+)	Low	Low	Multi-center data (BIDMC) with extensive external validation (UK Biobank).
Schoels et al. 2025 *[21]*	RoB: (+) App: (+)	RoB: (+) App: (+)	RoB: (+) App: (+)	RoB: (+)	Low	Low	Stroke unit patients externally validated on an independent multicenter RCT dataset (MonDAFIS).
Singh et al. 2022 *[22]*	RoB: (+) App: (+)	RoB: (+) App: (+)	RoB: (+) App: (+)	RoB: (+)	Low	Low	External validation present. Predicts AF occurrence within 15 days utilizing temporally separated 24h Holters.
Wu et al. 2024 *[23]*	RoB: (+) App: (+)	RoB: (+) App: (+)	RoB: (+) App: (+)	RoB: (+)	Low	Low	External validation performed at independent sites (Kameda General Hospital). Edge AI design increases clinical applicability.
Yuan et al. 2023 *[24]*	RoB: (-) App: (-)	RoB: (+) App: (+)	RoB: (+) App: (+)	RoB: (+)	High	High	Multi-site but heavily male-dominated (US Veterans cohort, ~94% male), raising high concerns for sex-based algorithmic bias and applicability.
Zeidaabadi et al. 2025 *[25]*	RoB: (+) App: (+)	RoB: (-) App: (-)	RoB: (+) App: (+)	RoB: (+)	High	High	Utilized scanned/PDF images. High applicability concern regarding whether models learn true physiological features or artifacts/compression noise from the image format itself.
Short-term AF onset							
Boon et al. 2016 *[26]*	RoB: (-) App: (-)	RoB: (+) App: (+)	RoB: (?) App: (?)	RoB: (-)	High	High	PhysioNet AFDB reliance.
Boon et al. 2018 *[27]*	RoB: (-) App: (-)	RoB: (+) App: (+)	RoB: (?) App: (?)	RoB: (-)	High	High	PhysioNet AFDB reliance. Identical limitations regarding class balance and spectrum bias.
Castro et al. 2021 *[28]*	RoB: (-) App: (-)	RoB: (+) App: (+)	RoB: (?) App: (?)	RoB: (-)	High	High	PhysioNet AFDB reliance.

Ebrahimzadeh et al. 2018 *[29]*	RoB: (-) App: (-)	RoB: (+) App: (+)	RoB: (?) App: (?)	RoB: (-)	High	High	PhysioNet AFDB reliance.
Grégoire et al. 2025 *[30]*	RoB: (+) App: (+)	RoB: (+) App: (+)	RoB: (+) App: (+)	RoB: (+)	Low	Low	Utilized a new, large Holter database overcoming the standard PhysioNet spectrum bias limitations for short-term prediction.
Mohebbi and Ghassemian 2012 *[31]*	RoB: (-) App: (-)	RoB: (+) App: (+)	RoB: (?) App: (?)	RoB: (-)	High	High	PhysioNet AFDB reliance. Overestimates real-world performance.
Narin et al. 2018 *[32]*	RoB: (-) App: (-)	RoB: (+) App: (+)	RoB: (?) App: (?)	RoB: (-)	High	High	Trained on PhysioNet AFDB. Artificial class balancing severely overestimates real-world positive predictive value.
Rooney et al. 2023 *[33]*	RoB: (-) App: (-)	RoB: (+) App: (+)	RoB: (+) App: (+)	RoB: (-)	High	High	Trained on overused open datasets (Physiobank). Lacks prospective clinical validation.

Note. RoB = Risk of Bias; App = Applicability; (+) = Low Risk/Concern; (-) = High Risk/Concern; (?) = Unclear Risk/Concern.

References

1. Tricco, A.C.; Lillie, E.; Zarin, W.; O'Brien, K.K.; Colquhoun, H.; Levac, D.; Moher, D.; Peters, M.D.J.; Horsley, T.; Weeks, L.; et al. PRISMA Extension for Scoping Reviews (PRISMA-ScR): Checklist and Explanation. *Ann. Intern. Med.* **2018**, *169*, 467–473, doi:10.7326/M18-0850.
2. Alreshidi, F.S.; Alsaffar, M.; Chengoden, R.; Alshammari, N.K. Fed-CL- an Atrial Fibrillation Prediction System Using ECG Signals Employing Federated Learning Mechanism. *Sci. Rep.* **2024**, *14*, 21038, doi:10.1038/s41598-024-71366-7.
3. Attia, Z.I.; Noseworthy, P.A.; Lopez-Jimenez, F.; Asirvatham, S.J.; Deshmukh, A.J.; Gersh, B.J.; Carter, R.E.; Yao, X.; Rabinstein, A.A.; Erickson, B.J.; et al. An Artificial Intelligence-Enabled ECG Algorithm for the Identification of Patients with Atrial Fibrillation during Sinus Rhythm: A Retrospective Analysis of Outcome Prediction. *The Lancet* **2019**, *394*, 861–867, doi:10.1016/S0140-6736(19)31721-0.
4. Baek, Y.-S.; Lee, S.-C.; Choi, W.; Kim, D.-H. A New Deep Learning Algorithm of 12-Lead Electrocardiogram for Identifying Atrial Fibrillation during Sinus Rhythm. *Sci. Rep.* **2021**, *11*, 12818, doi:10.1038/s41598-021-92172-5.
5. Brant, L.C.C.; Ribeiro, A.H.; Eromosele, O.B.; Pinto-Filho, M.M.; Barreto, S.M.; Duncan, B.B.; Larson, M.G.; Benjamin, E.J.; Ribeiro, A.L.P.; Lin, H. Prediction of Atrial Fibrillation From the ECG in the Community Using Deep Learning: A Multinational Study. *Circ. Arrhythm. Electrophysiol.* **2025**, *18*, doi:10.1161/CIRCEP.125.013734.
6. Cho, S.; Eom, S.; Kim, D.; Kim, T.-H.; Uhm, J.-S.; Pak, H.-N.; Lee, M.-H.; Yang, P.-S.; Lee, E.; Attia, Z.I.; et al. Artificial Intelligence–Derived Electrocardiographic Aging and Risk of Atrial Fibrillation: A Multi-National Study. *Eur. Heart J.* **2025**, *46*, 839–852, doi:10.1093/eurheartj/ehae790.
7. Choi, J.; Song, S.; Kim, H.; Kim, J.; Park, H.; Jeon, J.; Hong, J.; Gwag, H.B.; Lee, S.H.; Lee, J.; et al. Machine Learning Algorithm to Predict Atrial Fibrillation Using Serial 12-Lead ECGs Based on Left Atrial Remodeling. *J. Am. Heart Assoc.* **2024**, *13*, e034154, doi:10.1161/JAHA.123.034154.

8. Christopoulos, G.; Graff-Radford, J.; Lopez, C.L.; Yao, X.; Attia, Z.I.; Rabinstein, A.A.; Petersen, R.C.; Knopman, D.S.; Mielke, M.M.; Kremers, W.; et al. Artificial Intelligence–Electrocardiography to Predict Incident Atrial Fibrillation: A Population-Based Study. *Circ. Arrhythm. Electrophysiol.* **2020**, *13*, e009355, doi:10.1161/CIRCEP.120.009355.
9. Dupulthys, S.; Dujardin, K.; Anné, W.; Pollet, P.; Vanhaverbeke, M.; McAuliffe, D.; Lammertyn, P.-J.; Berteloot, L.; Mertens, N.; De Jaeger, P. Single-Lead Electrocardiogram Artificial Intelligence Model with Risk Factors Detects Atrial Fibrillation during Sinus Rhythm. *Europace* **2024**, *26*, euad354, doi:10.1093/europace/euad354.
10. Gadaleta, M.; Harrington, P.; Barnhill, E.; Hytopoulos, E.; Turakhia, M.P.; Steinhubl, S.R.; Quer, G. Prediction of Atrial Fibrillation from At-Home Single-Lead ECG Signals without Arrhythmias. *Npj Digit. Med.* **2023**, *6*, 229, doi:10.1038/s41746-023-00966-w.
11. Hygrell, T.; Viberg, F.; Dahlberg, E.; Charlton, P.H.; Kemp Gudmundsdottir, K.; Mant, J.; Hörnlund, J.L.; Svennberg, E. An Artificial Intelligence–Based Model for Prediction of Atrial Fibrillation from Single-Lead Sinus Rhythm Electrocardiograms Facilitating Screening. *EP Eur.* **2023**, *25*, 1332–1338, doi:10.1093/europace/euad036.
12. Jabbour, G.; Nolin-Lapalme, A.; Tastet, O.; Corbin, D.; Jordà, P.; Sowa, A.; Delfrate, J.; Busseuil, D.; Hussin, J.G.; Dubé, M.-P.; et al. Prediction of Incident Atrial Fibrillation Using Deep Learning, Clinical Models, and Polygenic Scores. *Eur. Heart J.* **2024**, *45*, 4920–4934, doi:10.1093/eurheartj/ehae595.
13. Khurshid, S.; Friedman, S.; Reeder, C.; Di Achille, P.; Diamant, N.; Singh, P.; Harrington, L.X.; Wang, X.; Al-Alusi, M.A.; Sarma, G.; et al. ECG-Based Deep Learning and Clinical Risk Factors to Predict Atrial Fibrillation. *Circulation* **2022**, *145*, 122–133, doi:10.1161/CIRCULATIONAHA.121.057480.
14. Kim, J.Y.; Kim, K.G.; Tae, Y.; Chang, M.; Park, S.-J.; Park, K.-M.; On, Y.K.; Kim, J.S.; Lee, Y.; Jang, S.-W. An Artificial Intelligence Algorithm With 24-h Holter Monitoring for the Identification of Occult Atrial Fibrillation During Sinus Rhythm. *Front. Cardiovasc. Med.* **2022**, *9*, 906780, doi:10.3389/fcvm.2022.906780.
15. Lee, J.H.; Kim, J.; Choi, J.; Choi, Y.Y.; Oh, I.-Y.; Cho, Y. Interethnic Validation of Artificial Intelligence for Prediction of Atrial Fibrillation Using Sinus Rhythm Electrocardiogram. *J. Cardiovasc. Med.* **2025**, *26*, 692–698, doi:10.2459/JCM.0000000000001798.
16. Melzi, P.; Vera-Rodriguez, R.; Tolosana, R.; Sanz-Garcia, A.; Cecconi, A.; Ortega, G.J.; Jimenez-Borreguero, L.J. Prediction of Atrial Fibrillation from Sinus-Rhythm Electrocardiograms Based on Deep Neural Networks: Analysis of Time Intervals and Longitudinal Study. *IRBM* **2023**, *44*, 100811, doi:10.1016/j.irbm.2023.100811.
17. Noseworthy, P.A.; Attia, Z.I.; Behnken, E.M.; Giblon, R.E.; Bews, K.A.; Liu, S.; Gosse, T.A.; Linn, Z.D.; Deng, Y.; Yin, J.; et al. Artificial Intelligence-Guided Screening for Atrial Fibrillation Using Electrocardiogram during Sinus Rhythm: A Prospective Non-Randomised Interventional Trial. *The Lancet* **2022**, *400*, 1206–1212, doi:10.1016/S0140-6736(22)01637-3.
18. Rabinstein, A.A.; Yost, M.D.; Faust, L.; Kashou, A.H.; Latif, O.S.; Graff-Radford, J.; Attia, I.Z.; Yao, X.; Noseworthy, P.A.; Friedman, P.A. Artificial Intelligence-Enabled ECG to Identify Silent Atrial Fibrillation in Embolic Stroke of Unknown Source. *J. Stroke Cerebrovasc. Dis.* **2021**, *30*, 105998, doi:10.1016/j.jstrokecerebrovasdis.2021.105998.
19. Raghunath, S.; Pfeifer, J.M.; Ulloa-Cerna, A.E.; Nemani, A.; Carbonati, T.; Jing, L.; vanMaanen, D.P.; Hartzel, D.N.; Ruhl, J.A.; Lagerman, B.F.; et al. Deep Neural Networks Can Predict New-Onset Atrial Fibrillation From the 12-Lead ECG and Help Identify Those at Risk of Atrial Fibrillation–Related Stroke. *Circulation* **2021**, *143*, 1287–1298, doi:10.1161/CIRCULATIONAHA.120.047829.
20. Sau, A.; Sieliwonczyk, E.; Barker, J.; Zeidaabadi, B.; Pastika, L.; Patlatzoglou, K.; Khattak, G.R.; McGurk, K.A.; Peters, N.S.; Kramer, D.B.; et al. Prediction of Incident Atrial Fibrillation: A Comprehensive Evaluation of Conventional and Artificial Intelligence-Enhanced Approaches. *Heart Rhythm* **2025**, S154752712502778X, doi:10.1016/j.hrthm.2025.08.024.
21. Schoels, M.; Krumm, L.; Nelde, A.; Olma, M.C.; Nolte, C.H.; Scheitz, J.F.; Klammer, M.G.; Leithner, C.; Meisel, A.; Scheibe, F.; et al. Artificial Intelligence for Prediction of Atrial Fibrillation in the Stroke Unit: A Retrospective Derivation Validation Cohort Study. *eBioMedicine* **2025**, *118*, 105869, doi:10.1016/j.ebiom.2025.105869.
22. Singh, J.P.; Fontanarava, J.; De Massé, G.; Carbonati, T.; Li, J.; Henry, C.; Fiorina, L. Short-Term Prediction of Atrial Fibrillation from Ambulatory Monitoring ECG Using a Deep Neural Network. *Eur. Heart J. - Digit. Health* **2022**, *3*, 208–217, doi:10.1093/ehjdh/ztac014.
23. Wu, H.; Sawada, T.; Goto, T.; Yoneyama, T.; Sasano, T.; Asada, K. Edge AI Model Deployed for Real-Time Detection of Atrial Fibrillation Risk during Sinus Rhythm. *J. Clin. Med.* **2024**, *13*, 2218, doi:10.3390/jcm13082218.
24. Yuan, N.; Duffy, G.; Dhruva, S.S.; Oesterle, A.; Pellegrini, C.N.; Theurer, J.; Vali, M.; Heidenreich, P.A.; Keyhani, S.; Ouyang, D. Deep Learning of Electrocardiograms in Sinus Rhythm From US Veterans to Predict Atrial Fibrillation. *JAMA Cardiol.* **2023**, *8*, 1131, doi:10.1001/jamacardio.2023.3701.
25. Zeidaabadi, B.; Patlatzoglou, K.; Barker, J.; Pastika, L.; Khattak, G.R.; Gurnani, M.; Da Silva Anjos Machado, X.; Peters, N.S.; Kramer, D.B.; Waks, J.W.; et al. Image Based Artificial Intelligence-Enhanced Electrocardiogram Prediction of Incident Atrial Fibrillation. *Heart Rhythm* **2025**, S1547527125029777, doi:10.1016/j.hrthm.2025.10.024.
26. Boon, K.H.; Khalil-Hani, M.; Malarvili, M.B.; Sia, C.W. Paroxysmal Atrial Fibrillation Prediction Method with Shorter HRV Sequences. *Comput. Methods Programs Biomed.* **2016**, *134*, 187–196, doi:10.1016/j.cmpb.2016.07.016.
27. Boon, K.H.; Khalil-Hani, M.; Malarvili, M. Paroxysmal Atrial Fibrillation Prediction Based on HRV Analysis and Non-Dominated Sorting Genetic Algorithm III. *Comput. Methods Programs Biomed.* **2018**, *153*, 171–184, doi:10.1016/j.cmpb.2017.10.012.
28. Castro, H.; Garcia-Racines, J.D.; Bernal-Norena, A. Methodology for the Prediction of Paroxysmal Atrial Fibrillation Based on Heart Rate Variability Feature Analysis. *Heliyon* **2021**, *7*, e08244, doi:10.1016/j.heliyon.2021.e08244.

29. Ebrahimzadeh, E.; Kalantari, M.; Joulani, M.; Shahraki, R.S.; Fayaz, F.; Ahmadi, F. Prediction of Paroxysmal Atrial Fibrillation: A Machine Learning Based Approach Using Combined Feature Vector and Mixture of Expert Classification on HRV Signal. *Comput. Methods Programs Biomed.* **2018**, *165*, 53–67, doi:10.1016/j.cmpb.2018.07.014.
30. Grégoire, J.-M.; Gilon, C.; Marelli, F.; Bersini, H.; Groben, L.; Nguyen, T.; Deruyter, B.; Godart, P.; Carlier, S. Short-Term Atrial Fibrillation Onset Prediction Using Machine Learning. *Eur. Heart J. - Digit. Health* **2025**, *6*, 1159–1168, doi:10.1093/ehjdh/ztaf104.
31. Mohebbi, M.; Ghassemian, H. Prediction of Paroxysmal Atrial Fibrillation Based on Non-Linear Analysis and Spectrum and Bispectrum Features of the Heart Rate Variability Signal. *Comput. Methods Programs Biomed.* **2012**, *105*, 40–49, doi:10.1016/j.cmpb.2010.07.011.
32. Narin, A.; Isler, Y.; Ozer, M.; Perc, M. Early Prediction of Paroxysmal Atrial Fibrillation Based on Short-Term Heart Rate Variability. *Phys. Stat. Mech. Its Appl.* **2018**, *509*, 56–65, doi:10.1016/j.physa.2018.06.022.
33. Rooney, S.R.; Kaufman, R.; Murugan, R.; Kashani, K.B.; Pinsky, M.R.; Al-Zaiti, S.; Dubrawski, A.; Clermont, G.; Miller, J.K. Forecasting Imminent Atrial Fibrillation in Long-Term Electrocardiogram Recordings. *J. Electrocardiol.* **2023**, *81*, 111–116, doi:10.1016/j.jelectrocard.2023.08.011.
